# Urinary Albumin-to-Creatinine Ratio as an Independent Predictor of 90-Day Outcomes in Patients Hospitalized for Acute Decompensated Heart Failure

**DOI:** 10.3390/jcm15072690

**Published:** 2026-04-02

**Authors:** Claudia Andreea Palcău, Livia Florentina Păduraru, Ana Maria Alexandra Stănescu

**Affiliations:** 1 Faculty of Medicine, “Carol Davila” University of Medicine and Pharmacy, 050474 Bucharest, Romania; claudia-andreea.nistor@drd.umfcd.ro (C.A.P.); alexandra.stanescu@umfcd.ro (A.M.A.S.); 2 Department of Cardiology, Elias University Hospital, 011461 Bucharest, Romania; 3 Department of Family Medicine, “Carol Davila” Central Military Emergency University Hospital, 010242 Bucharest, Romania; 4 Academy of Romanian Scientists (AOSR), 050085 Bucharest, Romania; 5 “Emil Palade” Center of Excellence for Young Researchers EP-CEYR The Academy of Romanian Scientists AOSR, 050085 Bucharest, Romania

**Keywords:** acute heart failure, albuminuria, NT-proBNP, renal dysfunction, cardiorenal syndrome, short-term outcomes, prognosis, risk stratification

## Abstract

**Background:** Albuminuria reflects systemic endothelial dysfunction and cardiorenal interaction in heart failure (HF), yet its short-term prognostic value in acute decompensated HF (ADHF) remains incompletely characterized. **Methods:** We conducted a prospective observational cohort study including 144 patients with complete follow-up hospitalized for ADHF. Urinary albumin-to-creatinine ratio (ACR), NT-proBNP, and estimated glomerular filtration rate (eGFR) were measured within 24 h of admission. Prior HF hospitalization within 12 months was recorded. The primary endpoint was a 90-day post-discharge composite of all-cause mortality or HF rehospitalization. Associations were examined using logistic regression, and discrimination was assessed using ROC curves with AUC comparisons. **Results:** Twenty-six patients (18.1%) experienced the 90-day composite endpoint. In univariable analysis, log_10_-transformed ACR was strongly associated with events (OR 3.90, 95% CI 1.92–7.91; *p* < 0.001). In multivariable analysis, ACR remained independently associated with the endpoint in Model 1 (ACR + prior HF hospitalization: OR 4.21, 95% CI 1.93–9.17; *p* < 0.001) and Model 2 (additional adjustment for log_10_ NT-proBNP: OR 3.49, 95% CI 1.54–7.91; *p* = 0.003). NT-proBNP was not independently associated with outcome in the fully adjusted model (*p* = 0.080). Discrimination improved from AUC 0.724 for ACR alone to 0.821 for Model 1 and 0.836 for Model 2; the AUC difference between Model 1 and Model 2 was not statistically significant (*p* = 0.404). **Conclusions:** Urinary ACR independently predicts 90-day adverse outcomes after ADHF hospitalization and improves discrimination when combined with recent HF hospitalization history; NT-proBNP did not provide significant incremental discrimination beyond this model.

## 1. Introduction

Heart failure (HF) remains a major global public health challenge, characterized by high rates of hospitalization, rehospitalization, and short-term mortality [1]. Patients hospitalized for acute decompensated heart failure (ADHF) represent a particularly vulnerable population, with a substantial risk of early adverse outcomes following discharge [2,3]. Accurate risk stratification during index hospitalization is therefore essential for guiding clinical decision-making and post-discharge management strategies.

Natriuretic peptides, particularly N-terminal pro-B-type natriuretic peptide (NT-proBNP), are well-established biomarkers reflecting myocardial wall stress and hemodynamic burden [4]. Elevated NT-proBNP levels are consistently associated with adverse prognosis in both chronic and acute HF settings [5]. NT-proBNP primarily reflects acute volume overload and ventricular strain, and it does not fully capture the broader pathophysiological processes underlying disease progression, including endothelial dysfunction, systemic inflammation, and renal involvement [6].

The interplay between cardiac and renal dysfunction is central to HF pathophysiology. The concept of the cardiorenal axis highlights the bidirectional relationship between impaired cardiac function and progressive renal injury [7]. In this context, albuminuria, quantified by the urinary albumin-to-creatinine ratio (ACR), represents a marker of glomerular barrier dysfunction and systemic endothelial injury [8]. Beyond reflecting renal damage, albuminuria has been associated with microvascular dysfunction, inflammation, and adverse cardiovascular outcomes across multiple clinical populations [9].

In patients with HF, albuminuria has been increasingly recognized as a potential prognostic marker. However, its independent and incremental predictive value in the setting of ADHF particularly in relation to established biomarkers, such as NT-proBNP and clinical severity markers, remains incompletely defined [10,11,12]. Moreover, the extent to which albuminuria improves short-term risk discrimination beyond conventional predictors requires further clarification [9].

Therefore, the present study aimed to evaluate the independent association between urinary ACR and 90-day post-discharge adverse outcomes in patients hospitalized for ADHF. Additionally, we sought to assess whether ACR provides incremental prognostic information beyond prior HF hospitalization and NT-proBNP levels.

## 2. Materials and Methods

### 2.1. Study Design and Population

**Figure 1:**
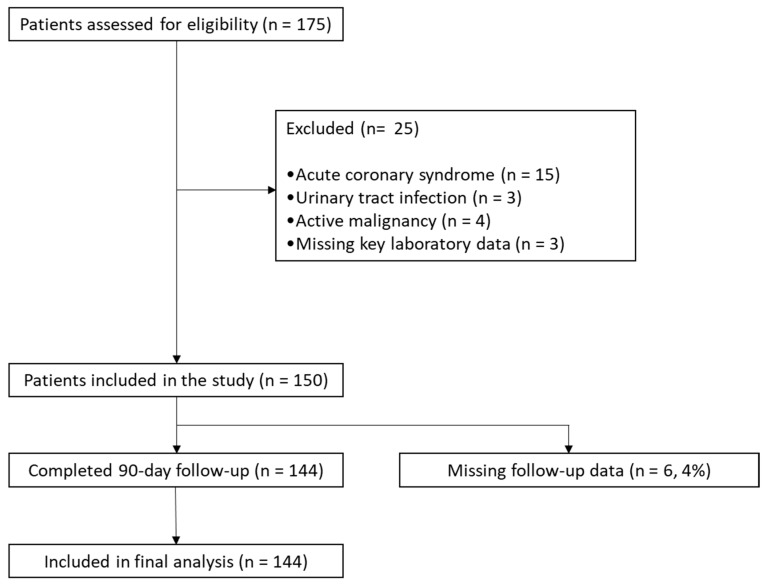
Study flow diagram.

We conducted a prospective, observational cohort study at the Elias University Hospital, department of Cardiology in Bucharest. A total of 175 patients with a primary diagnosis of ADHF were consecutively recruited during the index hospitalization between July 2025 and November 2025. Of the initially enrolled patients, 150 were included in the study cohort, of whom 144 (96.0%) completed the 90-day follow-up and were included in the final analysis, while 6 patients had missing follow-up data. HF was diagnosed according to current European Society of Cardiology (ESC) guidelines [13,14], based on clinical presentation, elevated natriuretic peptide levels, and echocardiographic evidence of structural and/or functional cardiac abnormalities. Echocardiography was performed using a high-resolution ultrasound system available in the cardiology department. Both patients with newly diagnosed HF and those with previously known HF were eligible for inclusion. Exclusion criteria included: acute coronary syndromes at presentation, acute urinary tract infection at the time of urine sampling, active malignancy and missing key laboratory data. The study was conducted in accordance with the Declaration of Helsinki and was approved by the local Ethics Committee (28072025-2/28 July 2025). A flow diagram summarizing patient selection and follow-up is presented in Figure 1.

### 2.2. Data Collection and Variable Definitions

Baseline demographic, clinical, laboratory, and echocardiographic data were collected during the index hospitalization using standardized case report forms. Recorded demographic and clinical variables included age, sex, smoking status, blood pressure, heart rate, New York Heart Association (NYHA) functional class at admission, and history of HF hospitalizations within the preceding 12 months.

Chronic outpatient pharmacological therapy prior to admission was documented from medical records and patient interviews. Recorded medications included beta-blockers, angiotensin-converting enzyme inhibitors (ACEIs), angiotensin receptor blockers (ARBs) or angiotensin receptor–neprilysin inhibitors (ARNIs), mineralocorticoid receptor antagonists (MRAs), sodium–glucose cotransporter 2 inhibitors (SGLT2is) and loop diuretics. The selected medication classes were chosen to reflect guideline-directed HF therapy and congestion management. Other concomitant medications were not systematically recorded, as they were outside the primary scope of the study.

HF phenotype was classified according to left ventricular ejection fraction (LVEF), assessed by transthoracic echocardiography during hospitalization, in accordance with current ESC criteria: HF with preserved ejection fraction (HFpEF, LVEF ≥ 50%), HF with mildly reduced ejection fraction (HFmrEF, LVEF 41–49%), and HF with reduced ejection fraction (HFrEF, LVEF ≤ 40%). LVEF was additionally recorded as a continuous variable at baseline.

Blood and urine samples were obtained within the first 24 h of hospital admission as part of routine clinical care. Laboratory analyses were performed in the hospital’s certified central laboratory using standardized automated assays. Measured parameters included serum creatinine, sodium, potassium, hemoglobin, leukocyte count, glucose, and C-reactive protein. Estimated glomerular filtration rate (eGFR) was calculated using the Chronic Kidney Disease Epidemiology Collaboration (CKD-EPI) equation.

Albuminuria was assessed using the urinary albumin-to-creatinine ratio (ACR), measured from a spot urine sample and expressed in mg/g. For descriptive analyses, albuminuria was categorized according to KDIGO [15] thresholds as A1 (<30 mg/g), A2 (30–300 mg/g), and A3 (>300 mg/g). N-terminal pro-B-type natriuretic peptide (NT-proBNP) concentrations were measured at admission using a standardized immunoassay. Due to right-skewed distributions, ACR and NT-proBNP values were logarithmically transformed (base 10) prior to inclusion in regression models.

### 2.3. Outcomes and Follow-Up

Patients were prospectively followed for 90 days after hospital discharge. Follow-up data were obtained through systematic review of hospital electronic medical records and, when necessary, direct telephone contact with patients or family members.

The primary endpoint was a composite of all-cause mortality or HF-related rehospitalization 90 days post-discharge. HF rehospitalization was defined as an unplanned hospital admission requiring intravenous diuretic therapy or escalation of HF-specific treatment in the setting of clinical signs and symptoms of congestion. All events were adjudicated based on available clinical documentation.

### 2.4. Statistical Analysis

Continuous variables were tested for normality using visual inspection of histograms and were expressed as median and interquartile range (IQR), given their non-normal distribution. Categorical variables were presented as counts and percentages. Between-group comparisons were performed using the Mann–Whitney U test for continuous variables and the chi-square test or Fisher’s exact test, as appropriate, for categorical variables. Correlations between continuous variables were assessed using Spearman’s rank correlation coefficient. Given the markedly right-skewed distributions of urinary albumin-to-creatinine ratio (ACR) and NT-proBNP, both variables were logarithmically transformed (base 10) prior to regression analyses.

Univariable logistic regression analyses were performed to identify predictors of the 90-day post-discharge composite endpoint. In the context of a limited number of outcome events, a parsimonious modeling strategy was adopted to avoid overfitting. Variables were selected based on clinical relevance and predefined study hypotheses, prioritizing albuminuria as the primary exposure of interest. The primary model (Model 1) included log_10_-transformed ACR and prior heart failure hospitalization within the preceding 12 months. A secondary model (Model 2) additionally included log_10_-transformed NT-proBNP to assess incremental prognostic value. Although additional covariates such as age, renal function (eGFR), and diabetes mellitus were evaluated in univariable analyses, they were not included in the final multivariable models in order to preserve model stability. Given the fixed follow-up period and the limited number of outcome events, logistic regression was preferred over time-to-event analysis. Analyses were performed on patients with complete follow-up data.

Multicollinearity among predictors was evaluated using variance inflation factors (VIFs), with VIF values < 5 considered acceptable. Model calibration was assessed using the Hosmer–Lemeshow goodness-of-fit test, and explanatory performance was evaluated using Nagelkerke’s R^2^. Discriminative ability was evaluated using receiver operating characteristic (ROC) curve analysis, and the area under the curve (AUC) was calculated for ACR alone and for the multivariable models. The optimal ACR cut-off was determined using the Youden Index derived from ROC curve analysis based on log_10_-transformed ACR values, with the corresponding threshold expressed in original ACR units (mg/g) for clinical interpretability. Results are reported as odds ratios (ORs) with 95% confidence intervals (CIs). A two-sided *p* value < 0.05 was considered statistically significant. Statistical analyses were performed using SPSS Statistics version 26 (IBM Corp., Armonk, NY, USA).

## 3. Results

### 3.1. Demographic and Clinical Characteristics

A total of 150 patients hospitalized with ADHF were enrolled in the study, of whom 144 (96%) completed the 90-day follow-up and were included in the final analysis. A total of 26 patients (18.1%) experienced the composite endpoint. In the overall cohort, chronic kidney disease (CKD) was present in 90 patients (62.5%), and diabetes mellitus in 68 patients (47.2%). HF phenotype distribution was as follows: HFpEF in 57 patients (39.6%), HFmrEF in 38 patients (26.4%), and HFrEF in 49 patients (34.0%). Baseline demographic characteristics were otherwise broadly comparable between groups. Age did not differ significantly, and sex distribution and smoking status were similar among patients with and without events. The prevalence of major cardiovascular comorbidities—including hypertension, diabetes mellitus, dyslipidemia, atrial fibrillation, and coronary heart disease—was also comparable between groups. Although CKD was more frequent among patients who reached the composite endpoint, this difference did not reach statistical significance.

Baseline LVEF was significantly lower among patients who experienced the composite endpoint compared with those without events (40% [23–45] vs. 45% [35–55], *p* = 0.009).

Regarding HF phenotype, HFrEF was more prevalent among patients with adverse outcomes (50.0% vs. 30.5%), whereas HFpEF was less common in this subgroup (19.2% vs. 44.1%), yielding a borderline overall association (*p* = 0.052).

Renal parameters differed significantly between groups. Patients who experienced the composite endpoint had lower eGFR values (median 46.3 vs. 67.6 mL/min/1.73 m^2^, *p* = 0.017) and higher serum creatinine levels (median 1.51 vs. 1.09 mg/dL, *p* = 0.039). In addition, albuminuria was markedly higher in the event group, with significantly elevated urinary albumin-to-creatinine ratio values (median 295.1 vs. 71.7 mg/g, *p* < 0.001). NT-proBNP concentrations were also substantially increased among patients who developed adverse outcomes (median 9577 vs. 3006 pg/mL, *p* < 0.001).

Markers of recent clinical instability showed significant between-group differences. Prior HF hospitalization within the preceding 12 months was more frequent among patients with events (57.7% vs. 17.8%, *p* < 0.001). Similarly, the need for non-invasive ventilation at admission was higher in this group (53.8% vs. 12.7%, *p* < 0.001).

Baseline medical therapy was largely comparable between groups. The use of guideline-directed HF therapies—including beta-blockers, ACEI/ARB/ARNI, mineralocorticoid receptor antagonists, and SGLT2 inhibitors—did not differ significantly. However, loop diuretics were more frequently prescribed at baseline in patients who subsequently experienced the composite endpoint (80.8% vs. 42.4%, *p* < 0.001).

Detailed results are presented in Table 1.

### 3.2. Univariate Logistic Regression Analysis

In univariate logistic regression analysis, markers of renal injury and neurohormonal activation were significantly associated with the 90-day post-discharge composite endpoint. Higher log_10_-transformed urinary albumin-to-creatinine ratio (ACR) was strongly associated with adverse outcomes (OR 3.90, 95% CI 1.92–7.91, *p* < 0.001). Likewise, elevated log_10_-transformed NT-proBNP levels were independently associated with increased risk (OR 4.80, 95% CI 1.93–11.91, *p* = 0.001).

Renal filtration parameters were also significantly related to outcome. Lower estimated glomerular filtration rate (eGFR) predicted a higher likelihood of events (OR 0.981 per 1 mL/min/1.73 m^2^ increase, 95% CI 0.965–0.997, *p* = 0.022), corresponding to a 1.9% reduction in event odds for each incremental increase in eGFR.

In addition, HF phenotype and prior HF hospitalization were associated with the endpoint in univariable analysis. Compared with HFrEF, HFpEF was associated with lower event odds (OR 0.27, 95% CI 0.09–0.81, *p* = 0.020), whereas prior HF hospitalization was associated with increased risk (OR 2.34, 95% CI 1.41–3.89, *p* < 0.001).

Detailed results are presented in Table 2.

### 3.3. Multivariable Logistic Regression Analysis

Multivariable logistic regression analysis identified ACR and prior HF hospitalization as independent predictors of the 90-day post-discharge composite endpoint.

In Model 1, including log_10_-transformed ACR and prior heart failure hospitalization within the preceding 12 months, both variables remained independently associated with adverse outcomes. Higher ACR levels were significantly associated with increased risk (OR 4.21, 95% CI 1.93–9.17, *p* < 0.001). Similarly, a history of at least one HF hospitalization during the prior year was a strong independent predictor (OR 6.72, 95% CI 2.48–18.23, *p* < 0.001). The model demonstrated adequate calibration (Hosmer–Lemeshow *p* = 0.264) and moderate explanatory capacity (Nagelkerke R^2^ = 0.321).

To further assess the contribution of neurohormonal activation, Model 2 incorporated log_10_-transformed NT-proBNP in addition to ACR and prior HF hospitalization. In this extended model, ACR remained independently associated with the composite endpoint (OR 3.49, 95% CI 1.54–7.91, *p* = 0.003), and prior HF hospitalization retained statistical significance. In contrast, NT-proBNP was not independently associated with outcome after adjustment (OR 2.50, 95% CI 0.90–6.99, *p* = 0.080). Model 2 showed comparable calibration (Hosmer–Lemeshow *p* = 0.251) and a modest increase in explanatory performance (Nagelkerke R^2^ = 0.351).

Collinearity diagnostics revealed no evidence of multicollinearity among predictors, with all variance inflation factors below 1.3.

Detailed results are presented in Table 3.

### 3.4. Discriminative Performance and ROC Analysis

Receiver operating characteristic (ROC) curve analysis was performed to evaluate the discriminative performance of log_10_-transformed albumin-to-creatinine ratio (ACR) and the multivariable models for prediction of the 90-day post-discharge composite endpoint.

Log_10_-transformed ACR alone demonstrated acceptable discrimination, with an area under the curve (AUC) of 0.724. The addition of prior heart failure hospitalization (Model 1) improved discriminative performance, yielding an AUC of 0.821. When log_10_-transformed NT-proBNP was further incorporated (Model 2), the AUC increased modestly to 0.836.

Pairwise ROC curve comparisons showed that Model 1 significantly improved discrimination compared with ACR alone (*p* = 0.028). Likewise, Model 2 demonstrated significantly higher discrimination than ACR alone (*p* = 0.011). However, the difference between Model 1 and Model 2 was not statistically significant (*p* = 0.404).

The optimal ACR threshold identified using the Youden Index (derived from ROC analysis of log_10_-transformed ACR) was 103 mg/g, yielding a sensitivity of 76.9% and a specificity of 61.9% for the prediction of the 90-day post-discharge endpoint.

**Figure 2:**
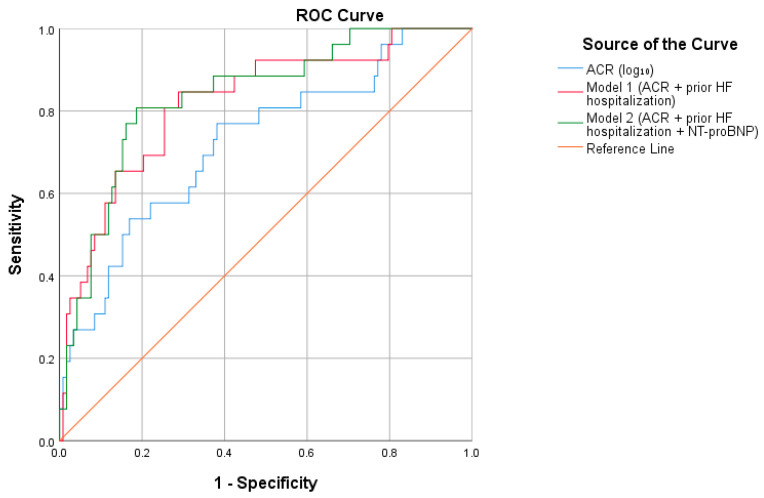
Receiver operating characteristic (ROC) curves evaluating the discriminative performance of log_10_-transformed albumin-to-creatinine ratio (ACR), Model 1 (ACR + previous heart failure hospitalization), and Model 2 (ACR + previous heart failure hospitalization + NT-proBNP) for the prediction of the 90-day post-discharge composite outcome.

The ROC curves for ACR alone, Model 1, and Model 2 are presented in Figure 2.

## 4. Discussion

In this prospective cohort of patients hospitalized for ADHF, albuminuria—quantified as log_10_-transformed urinary albumin-to-creatinine ratio (ACR)—emerged as a strong and independent predictor of short-term adverse outcomes. ACR remained independently associated with the 90-day post-discharge composite endpoint after adjustment for prior HF hospitalization and NT-proBNP levels. Importantly, the addition of prior HF hospitalization significantly improved model discrimination compared with ACR alone, while further inclusion of NT-proBNP did not provide statistically significant incremental prognostic value. These findings underscore the central role of renal injury markers and recent clinical instability in early risk stratification following ADHF hospitalization.

In the primary multivariable model, both ACR and prior HF hospitalization independently predicted adverse outcomes. A history of recent hospitalization likely reflects cumulative disease burden, advanced cardiac remodeling, and persistent hemodynamic vulnerability, serving as a clinical surrogate of instability and incomplete recovery after previous decompensations. In our cohort, prior HF hospitalization conferred an approximately six-fold higher risk of early adverse outcomes, reinforcing the well-established prognostic weight of recent clinical instability reported in previous observational studies [16,17,18].

Albuminuria, however, appears to capture a distinct biological dimension. Unlike glomerular filtration rate (eGFR), which reflects global filtration capacity, albuminuria indicates structural and functional alterations of the glomerular filtration barrier and systemic endothelial dysfunction [19,20]. Its persistence as an independent predictor after multivariable adjustment suggests that microvascular injury and chronic cardiorenal stress may be more prognostically informative than filtration impairment alone in the acute setting.

Although reduced eGFR was associated with adverse outcomes in univariable analysis, it did not retain independent significance after adjustment for albuminuria, aligning with growing evidence that albuminuria better reflects the active component of cardiorenal interaction—particularly endothelial injury and inflammatory activation—compared with static measures of renal filtration [8,21,22].

When NT-proBNP was incorporated into the extended model, ACR remained statistically significant, whereas NT-proBNP did not independently predict the composite endpoint. Collinearity diagnostics excluded significant multicollinearity, suggesting that the attenuation of NT-proBNP reflects overlapping pathophysiological information rather than statistical redundancy.

While NT-proBNP primarily reflects acute myocardial wall stress, volume overload, and neurohormonal activation at the time of decompensation [4], albuminuria reflects chronic endothelial dysfunction, microvascular injury, systemic inflammation, and persistent renal involvement—core components of the cardio-renal axis [23,24]. Beyond endothelial dysfunction, several additional mechanisms may contribute to the association between albuminuria and adverse outcomes in ADHF. Renal congestion, a hallmark of acute decompensation, leads to increased renal venous pressure and impaired glomerular filtration barrier integrity, promoting albumin leakage. Furthermore, activation of the cardiorenal axis results in a complex interplay between hemodynamic, neurohormonal, and inflammatory pathways, further exacerbating both cardiac and renal dysfunction. Systemic inflammation and microvascular dysfunction may additionally contribute to increased vascular permeability and progressive organ injury. This mechanistic distinction may explain why albuminuria retained independent prognostic value after adjustment, whereas NT-proBNP did not demonstrate significant incremental discrimination once clinical instability and renal injury were accounted for.

ROC curve analysis further supported the prognostic relevance of albuminuria. ACR alone demonstrated acceptable discrimination (AUC 0.724), and the addition of prior HF hospitalization significantly improved predictive performance (AUC 0.821; *p* = 0.028 vs. ACR alone). Although inclusion of NT-proBNP numerically increased the AUC to 0.836, pairwise comparison revealed no statistically significant difference between the two multivariable models (*p* = 0.404). These results suggest that integrating a marker of renal microvascular injury with a clinical indicator of recent instability meaningfully enhances early risk stratification, whereas additional neurohormonal biomarkers may offer limited incremental discrimination in this specific context.

Our findings are consistent with prior studies demonstrating an association between albuminuria and adverse outcomes in chronic HF populations, including both HFrEF and HFpEF phenotypes, where increasing levels of albuminuria are linked to stepwise increases in mortality risk independent of traditional renal function measures [12,25,26]. These observations are further supported by larger cohort and registry-based analyses, which consistently demonstrate that albuminuria is associated with increased mortality and hospitalization risk across the spectrum of HF, independent of conventional renal function parameters. In particular, studies such as that by Boorsma et al. have highlighted albuminuria as a marker of systemic congestion and vascular dysfunction, reinforcing its role as an integrative biomarker of cardiorenal interaction [11]. Similarly, data from acute heart failure cohorts, including the study by Matsumoto et al., have shown that elevated urinary ACR at admission is associated with early rehospitalization risk [27], supporting its relevance in short-term prognostic assessment.

Together, these findings support the concept that albuminuria reflects a pathophysiological axis that integrates hemodynamic stress, endothelial dysfunction, and renal injury.

However, data specifically addressing short-term outcomes following hospitalization for ADHF remain limited [27]. By focusing on the early post-discharge period, our study extends previous observations and suggests that albuminuria may be particularly relevant for identifying patients at heightened risk shortly after an episode of acute decompensation. The lack of significant incremental discrimination with NT-proBNP after multivariable adjustment, despite its well-established diagnostic utility [28], further highlights the important distinction between diagnostic and prognostic biomarkers in acute HF and underscores the multifactorial nature of early post-discharge risk.

From a clinical perspective, urinary ACR represents a simple, inexpensive, and widely available biomarker that may enhance early risk stratification in patients hospitalized with ADHF. Although NT-proBNP is routinely incorporated into acute HF management, albuminuria is not systematically included in inpatient prognostic assessment. The improvement in model discrimination from an AUC of 0.724 (ACR alone) to 0.821 with the addition of prior HF hospitalization emphasizes the importance of integrating biological markers of organ injury with indicators of clinical instability. Routine assessment of albuminuria at admission could facilitate identification of patients at higher risk for early adverse outcomes and may inform closer follow-up strategies or more intensive optimization of guideline-directed therapy in the vulnerable post-discharge period.

### Limitations

Several limitations should be acknowledged. First, this was a single-center study with a relatively small sample size, which may limit external validity and generalizability. Second, the follow-up period was restricted to 90 days; therefore, long-term prognostic implications remain uncertain. Third, although multivariable adjustment was performed, residual confounding cannot be excluded. Fourth, albuminuria was assessed at a single time point during the index hospitalization, and dynamic changes over time were not evaluated. A small proportion of patients (n = 6, <5%) had missing 90-day follow-up data. Given the low number of cases, a complete-case analysis was performed; however, the possibility that missingness was not completely at random cannot be entirely excluded. Finally, renal dysfunction may have influenced albuminuria levels, and the models were not externally validated in an independent cohort.

## 5. Conclusions

Albuminuria, assessed through the urinary albumin-to-creatinine ratio, was a strong and independent predictor of short-term adverse outcomes in patients hospitalized for ADHF. Its prognostic value remained significant after adjustment for prior HF hospitalization and NT-proBNP levels, suggesting that albuminuria captures clinically relevant pathophysiological information beyond traditional hemodynamic biomarkers.

Incorporation of urinary ACR into routine risk assessment may improve early risk stratification and help identify patients at increased risk for early post-discharge events. However, these findings should be confirmed in larger, multicenter studies before routine clinical implementation.

## Figures and Tables

**Table 1:** Baseline characteristics of the study population according to the 90-day post-discharge composite endpoint.

Variable	Total (n = 144)	No Event (n = 118)	Event (n = 26)	*p* Value
Demographic and clinical characteristics				
Age, years, median (IQR)		73.5 (65–82)	74.0 (65.8–82.8)	0.514
Male sex, n (%)	70 (48.6)	57 (48.3)	13 (50)	0.876
BMI category (obese), n (%)	80 (55.6)	68 (57.6)	12 (46.2)	0.287
Smoking (former/current), n (%)	66 (45.8)	56 (47.5)	10 (38.5)	0.405
Comorbidities				
Hypertension, n (%)	126 (87.5)	103 (87.3)	23 (88.5)	0.870
Dyslipidemia, n (%)	138 (95.8)	113 (95.8)	25 (96.2)	0.928
Diabetes mellitus, n (%)	68 (47.2)	55 (46.6)	13 (50.0)	0.754
Chronic kidney disease, n (%)	90 (62.5)	70 (59.3)	20 (76.9)	0.093
Atrial fibrillation, n (%)	79 (54.9)	64 (54.2)	15 (57.7)	0.749
Coronary heart disease, n (%)	63 (43.8)	50 (42.4)	13 (50)	0.478
Renal, biomarker, and echocardiographic parameters				
Serum creatinine, mg/dL, median (IQR)		1.09 (0.60–1.55)	1.51 (0.98–2.49)	0.039
eGFR (CKD-EPI), mL/min/1.73 m^2^, median (IQR)		67.6 (49.8–90.4)	46.3 (33.6–82.7)	0.017
Albumin-to-creatinine ratio, mg/g, median (IQR)		71.5 (30.1–181.3)	295.14 (95.9–993.6)	<0.001
NT-proBNP, pg/mL, median (IQR)		3006 (1212–9400)	9577 (4556–26,514)	<0.001
LVEF, %, median (IQR)		45 (35–55)	40 (23–45)	0.009
HF phenotype, n (%)				0.052
HFpEF	57 (39.6)	52 (44.1)	5 (19.2)	
HFmrEF	38 (26.4)	30 (25.4)	8 (30.8)	
HFrEF	49 (34.0)	36 (30.5)	13 (50.0)	
Previous HF hospitalization ≥ 1 in last 12 months, n (%)	36 (25.0)	21 (17.8)	15 (57.7)	<0.001
Ischemic HF, n (%)	61 (42.4)	48 (40.7)	13 (50.0)	0.384
NIV at admission, n (%)	29 (20.1)	15 (12.7)	14 (53.8)	0.001
Baseline medication				
Beta-blocker, n (%)	107 (74.3)	85 (72.0)	22 (84.6)	0.184
ACEI/ARB/ARNI, n (%)	108 (75.0)	89 (75.4)	19 (73.1)	0.802
MRA, n (%)	50 (34.7)	40 (33.9)	10 (38.5)	0.658
SGLT2i, n (%)	50 (34.7)	43 (36.4)	7 (26.9)	0.356
Loop diuretic, n (%)	71 (49.3)	50 (42.4)	21 (80.8)	<0.001

Abbreviations: BMI, body mass index; HF, heart failure; HFpEF, heart failure with preserved ejection fraction; HFmrEF, heart failure with mildly reduced ejection fraction; HFrEF, heart failure with reduced ejection fraction; LVEF, left ventricular ejection fraction; eGFR, estimated glomerular filtration rate; NT-proBNP, N-terminal pro-B-type natriuretic peptide; ACEI, angiotensin-converting enzyme inhibitors; ARB, angiotensin receptor blockers; ARNI, angiotensin receptor–neprilysin inhibitors; MRA, mineralocorticoid receptor antagonists; SGLT2i, sodium–glucose cotransporter 2 inhibitors; NIV, non-invasive ventilation.

**Table 2:** Univariate logistic regression analysis for the 90-day post-discharge composite outcome.

Variable	OR	95% CI	*p*-Value
ACR (log_10_)	3.90	1.92–7.91	<0.001
NT-proBNP (log_10_)	4.80	1.93–11.91	<0.001
eGFR (per 1 mL/min/1.73 m^2^ increase)	0.981	0.965–0.997	0.022
HFpEF vs. HFrEF	0.27	0.09–0.81	0.020
HFmrEF vs. HFrEF	0.74	0.27–2.02	0.554
Previous HF hospitalization	2.34	1.41–3.89	<0.001

ACR and NT-proBNP were log_10_-transformed due to right-skewed distributions. eGFR was analyzed as a continuous variable (per 1 mL/min/1.73 m^2^ increase). Reference category for heart failure phenotype: HFrEF. Abbreviations: ACR, albumin-to-creatinine ratio; NT-proBNP, N-terminal pro-B-type natriuretic peptide; eGFR, estimated glomerular filtration rate; HFpEF, heart failure with preserved ejection fraction; HFmrEF, heart failure with mildly reduced ejection fraction; HFrEF, heart failure with reduced ejection fraction; OR, odds ratio; CI, confidence interval.

**Table 3:** Multivariable logistic regression analysis for predictors of the 90-day post-discharge composite outcome.

Variable	Multivariate Model 1 OR (95% CI)	*p*	Multivariate Model 2 OR (95% CI)	*p*
ACR (log_10_)	4.21 (1.93–9.17)	<0.001	3.49 (1.54–7.91)	0.003
Previous HF hospitalization	6.72 (2.48–18.23)	<0.001	5.87 (2.12–16.26)	0.001
NT-proBNP (log_10_)	–	–	2.50 (0.90–6.99)	0.08

ACR and NT-proBNP were log_10_-transformed prior to analysis. Hosmer–Lemeshow test was used to assess model calibration. Nagelkerke R^2^ is reported as a measure of model explanatory power. Abbreviations: ACR, albumin-to-creatinine ratio; NT-proBNP, N-terminal pro-B-type natriuretic peptide; OR, odds ratio; CI, confidence interval.

## Data Availability

The data presented in this study are available on reasonable request from the corresponding author. The data are not publicly available due to privacy and ethical restrictions.
